# 2,2′-{[4,6-Bis(octyl­amino)-1,3,5-triazin-2-yl]aza­nedi­yl}diethanol

**DOI:** 10.1107/S1600536812003856

**Published:** 2012-02-04

**Authors:** Hong-Hua Sun, Zhi-Yong Hu, Duan-Lin Cao

**Affiliations:** a School of Chemical Engineering and Environment, North University of China, Taiyuan, People’s Republic ofChina

## Abstract

In the title compound, C_23_H_46_N_6_O_2_, the two hy­droxy groups are located on opposite sides of the triazine ring. One of the hy­droxy groups links with the triazine N atom *via* an intra­molecular O—H⋯N hydrogen bond. Inter­molecular O—H⋯N and N—H⋯O hydrogen bonding is observed in the crystal structure. π–π stacking is also observed between parallel triazine rings of adjacent mol­ecules, the centroid–centroid distance being 3.5944 (14) Å.

## Related literature

For the properties of Gemini surfacta­nts, see: Zana & Xia (2003 ▶); Menger & Keiper (2000 ▶). For the synthesis, see: Li *et al.* (2010 ▶); Zhao *et al.* (2010 ▶); Xue *et al.* (2011 ▶).
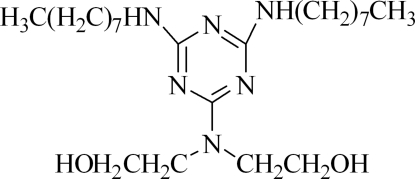


## Experimental

### 

#### Crystal data

C_23_H_46_N_6_O_2_
*M* *_r_* = 438.66
Triclinic, 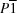

*a* = 8.333 (3) Å
*b* = 9.526 (3) Å
*c* = 17.182 (6) Å
α = 100.144 (7)°
β = 100.741 (5)°
γ = 102.109 (5)°
*V* = 1276.9 (8) Å^3^
*Z* = 2
Mo *K*α radiation
μ = 0.08 mm^−1^
*T* = 113 K
0.24 × 0.22 × 0.14 mm

#### Data collection

Rigaku Saturn724 CCD diffractometer
Absorption correction: multi-scan (*CrystalClear*; Rigaku/MSC, 2000 ▶) *T*_min_ = 0.982, *T*_max_ = 0.990
13414 measured reflections
6014 independent reflections
3623 reflections with *I* > 2σ(*I*)
*R*_int_ = 0.032

#### Refinement

*R*[*F* ^2^ > 2σ(*F* ^2^)] = 0.035
*wR*(*F* ^2^) = 0.092
*S* = 1.07
6014 reflections
292 parameters
2 restraints
H atoms treated by a mixture of independent and constrained refinement
Δρ_max_ = 0.22 e Å^−3^
Δρ_min_ = −0.35 e Å^−3^

### 

Data collection: *CrystalClear* (Rigaku/MSC, 2000 ▶); cell refinement: *CrystalClear*; data reduction: *CrystalClear*; program(s) used to solve structure: *SHELXTL* (Sheldrick, 2008 ▶); program(s) used to refine structure: *SHELXTL*; molecular graphics: *SHELXTL*; software used to prepare material for publication: *SHELXTL*.

## Supplementary Material

Crystal structure: contains datablock(s) I, global. DOI: 10.1107/S1600536812003856/xu5451sup1.cif

Structure factors: contains datablock(s) I. DOI: 10.1107/S1600536812003856/xu5451Isup2.hkl

Supplementary material file. DOI: 10.1107/S1600536812003856/xu5451Isup3.cml

Additional supplementary materials:  crystallographic information; 3D view; checkCIF report

## Figures and Tables

**Table 1:** Hydrogen-bond geometry (Å, °)

*D*—H⋯*A*	*D*—H	H⋯*A*	*D*⋯*A*	*D*—H⋯*A*
O1—H1⋯N2^i^	0.84	1.95	2.7732 (13)	167
O2—H2⋯N3	0.84	2.00	2.8057 (14)	160
N5—H5⋯O1^ii^	0.89 (1)	1.95 (1)	2.7963 (15)	157 (1)
N6—H6⋯O2^iii^	0.90 (1)	2.20 (1)	2.9455 (14)	141 (1)

Symmetry codes: (i) 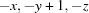; (ii) 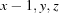; (iii) 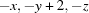.

**Fig. 1.**
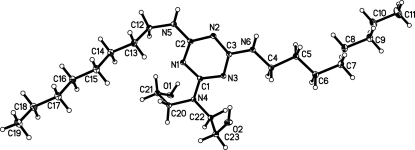
The molecular structure of the title compound with the atom numbering scheme. Displacement ellipsoids are drawn at the 30% probability level.

**Fig. 2.**
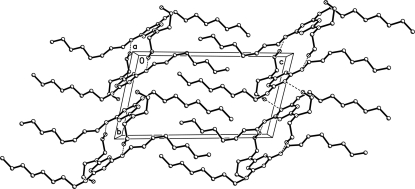
The crystal packing of the title compound.

## supplementary crystallographic information

### Comment

Recently, the majority of Gemini surfactants had been synthesized, because it shows much lower critical micelle concentration values, greater efficiency in lowering the surface tension of water, and interfacial tension at the oil/water interface than for the conventional surfactants (Zana & Xia, 2003; Menger & Keiper, 2000). The title compound, which can be prepared by the nucleophilic substitution reactions of 1-octylamine, diethanolamine and 2,4,6-trichloro-1,3,5-triazine. Similar this synthesis, see (Li *et al.*, 2010; Zhao *et al.*, 2010; Xue *et al.*, 2011). Here the crystal structure of the title compound is reported.

In the title molecule (Fig.1), there are two octylamino groups, two ethanol groups and a central triazin ring. In the crystal structure, the neighboring molecules are connected through weak intermolecular N—H···O and O—H···N interactions

### Experimental

The title compound was prepared according to literature method (Xue *et al.*, 2011). Single crystals suitable for X-ray diffraction were obtained by evaporation of a solution of the title compound in toluene at room temperature.

### Refinement

H atoms were positioned geometrically and treated as riding, with C—H = 0.98 for methyl H and 0.99 Å for methylene H atoms, O—H = 0.84 Å, and refined in riding mode with *U*_iso_(H) = 1.2*U*_eq_(C) for methylene H atoms and 1.5*U*_eq_(C,O) for methyl H and hydroxy H atoms. Imino H atoms were located in a difference Fourier map and refined isotroipically.

### Crystal data

**Table:**

C_23_H_46_N_6_O_2_	*Z* = 2
*M**_r_* = 438.66	*F*(000) = 484
Triclinic, *P*1	*D*_x_ = 1.141 Mg m^−^^3^
Hall symbol: -P 1	Mo *K*α radiation, λ = 0.71073 Å
*a* = 8.333 (3) Å	Cell parameters from 4402 reflections
*b* = 9.526 (3) Å	θ = 1.2–27.9°
*c* = 17.182 (6) Å	µ = 0.08 mm^−^^1^
α = 100.144 (7)°	*T* = 113 K
β = 100.741 (5)°	Prism, colorless
γ = 102.109 (5)°	0.24 × 0.22 × 0.14 mm
*V* = 1276.9 (8) Å^3^	

### Data collection

**Table:**

Rigaku Saturn724 CCD diffractometer	6014 independent reflections
Radiation source: rotating anode	3623 reflections with *I* > 2σ(*I*)
Multilayer monochromator	*R*_int_ = 0.032
Detector resolution: 14.22 pixels mm^-1^	θ_max_ = 27.9°, θ_min_ = 1.2°
ω and φ scans	*h* = −10→10
Absorption correction: multi-scan (*CrystalClear*; Rigaku/MSC, 2000)	*k* = −12→11
*T*_min_ = 0.982, *T*_max_ = 0.990	*l* = −22→22
13414 measured reflections	

### Refinement

**Table:**

Refinement on *F*^2^	Primary atom site location: structure-invariant direct methods
Least-squares matrix: full	Secondary atom site location: difference Fourier map
*R*[*F*^2^ > 2σ(*F*^2^)] = 0.035	Hydrogen site location: inferred from neighbouring sites
*wR*(*F*^2^) = 0.092	H atoms treated by a mixture of independent and constrained refinement
*S* = 1.07	*w* = 1/[σ^2^(*F*_o_^2^) + (0.034*P*)^2^] where *P* = (*F*_o_^2^ + 2*F*_c_^2^)/3
6014 reflections	(Δ/σ)_max_ = 0.003
292 parameters	Δρ_max_ = 0.22 e Å^−^^3^
2 restraints	Δρ_min_ = −0.35 e Å^−^^3^

### Special details

**Table:**

Geometry. All e.s.d.'s (except the e.s.d. in the dihedral angle between two l.s. planes) are estimated using the full covariance matrix. The cell e.s.d.'s are taken into account individually in the estimation of e.s.d.'s in distances, angles and torsion angles; correlations between e.s.d.'s in cell parameters are only used when they are defined by crystal symmetry. An approximate (isotropic) treatment of cell e.s.d.'s is used for estimating e.s.d.'s involving l.s. planes.
Refinement. Refinement of *F*^2^ against ALL reflections. The weighted *R*-factor *wR* and goodness of fit *S* are based on *F*^2^, conventional *R*-factors *R* are based on *F*, with *F* set to zero for negative *F*^2^. The threshold expression of *F*^2^ > σ(*F*^2^) is used only for calculating *R*-factors(gt) *etc*. and is not relevant to the choice of reflections for refinement. *R*-factors based on *F*^2^ are statistically about twice as large as those based on *F*, and *R*- factors based on ALL data will be even larger.

### Fractional atomic coordinates and isotropic or equivalent isotropic displacement parameters (Å^2^)

**Table:**

	*x*	*y*	*z*	*U*_iso_*/*U*_eq_	
O1	0.45621 (9)	0.45821 (9)	0.09768 (5)	0.0232 (2)	
H1	0.3754	0.4185	0.0572	0.035*	
O2	0.37413 (10)	1.04004 (9)	0.07962 (5)	0.0248 (2)	
H2	0.2881	0.9738	0.0533	0.037*	
N1	0.07742 (11)	0.62558 (10)	0.10758 (5)	0.0164 (2)	
N2	−0.15593 (11)	0.66811 (10)	0.01779 (6)	0.0161 (2)	
N3	0.12763 (11)	0.77449 (10)	0.01128 (5)	0.0156 (2)	
N4	0.34670 (11)	0.72741 (10)	0.09900 (6)	0.0168 (2)	
N5	−0.19488 (12)	0.52780 (11)	0.11208 (6)	0.0186 (2)	
N6	−0.10013 (11)	0.80796 (10)	−0.07507 (6)	0.0170 (2)	
C1	0.17873 (13)	0.70736 (12)	0.07153 (7)	0.0153 (2)	
C2	−0.08806 (13)	0.60962 (12)	0.07829 (7)	0.0154 (2)	
C3	−0.04067 (13)	0.74848 (12)	−0.01343 (7)	0.0153 (2)	
C4	0.00887 (13)	0.89922 (12)	−0.11373 (7)	0.0171 (3)	
H4A	0.1042	0.8556	−0.1216	0.021*	
H4B	0.0560	0.9986	−0.0777	0.021*	
C5	−0.08616 (14)	0.91258 (13)	−0.19511 (7)	0.0208 (3)	
H5A	−0.1813	0.9564	−0.1871	0.025*	
H5B	−0.1338	0.8131	−0.2309	0.025*	
C6	0.02670 (15)	1.00745 (13)	−0.23645 (7)	0.0232 (3)	
H6A	0.1174	0.9597	−0.2474	0.028*	
H6B	0.0807	1.1043	−0.1987	0.028*	
C7	−0.06691 (15)	1.03229 (13)	−0.31604 (7)	0.0247 (3)	
H7A	−0.1633	1.0722	−0.3055	0.030*	
H7B	0.0101	1.1081	−0.3337	0.030*	
C8	−0.13314 (15)	0.89670 (13)	−0.38531 (7)	0.0250 (3)	
H8A	−0.2097	0.8200	−0.3681	0.030*	
H8B	−0.0372	0.8574	−0.3971	0.030*	
C9	−0.22769 (16)	0.92779 (14)	−0.46281 (7)	0.0271 (3)	
H9A	−0.3247	0.9655	−0.4510	0.032*	
H9B	−0.1516	1.0061	−0.4792	0.032*	
C10	−0.29184 (17)	0.79495 (15)	−0.53305 (7)	0.0326 (3)	
H10A	−0.3668	0.7162	−0.5165	0.039*	
H10B	−0.1947	0.7580	−0.5453	0.039*	
C11	−0.3879 (2)	0.82560 (18)	−0.61002 (8)	0.0474 (4)	
H11A	−0.4866	0.8591	−0.5991	0.071*	
H11B	−0.4249	0.7353	−0.6530	0.071*	
H11C	−0.3141	0.9022	−0.6276	0.071*	
C12	−0.13547 (14)	0.45555 (13)	0.17567 (7)	0.0196 (3)	
H12A	−0.0466	0.4090	0.1607	0.023*	
H12B	−0.2301	0.3765	0.1795	0.023*	
C13	−0.06510 (14)	0.56152 (13)	0.25832 (7)	0.0213 (3)	
H13A	−0.1602	0.5878	0.2794	0.026*	
H13B	0.0068	0.6531	0.2512	0.026*	
C14	0.03814 (15)	0.50118 (13)	0.32134 (7)	0.0228 (3)	
H14A	−0.0347	0.4129	0.3313	0.027*	
H14B	0.1309	0.4708	0.2999	0.027*	
C15	0.11192 (15)	0.61478 (13)	0.40109 (7)	0.0248 (3)	
H15A	0.1833	0.7027	0.3900	0.030*	
H15B	0.0179	0.6454	0.4212	0.030*	
C16	0.21685 (15)	0.56546 (13)	0.46839 (7)	0.0240 (3)	
H16A	0.3141	0.5383	0.4499	0.029*	
H16B	0.1474	0.4766	0.4795	0.029*	
C17	0.28156 (15)	0.68470 (14)	0.54663 (7)	0.0249 (3)	
H17A	0.3520	0.7724	0.5350	0.030*	
H17B	0.1836	0.7135	0.5635	0.030*	
C18	0.38433 (16)	0.64197 (14)	0.61749 (7)	0.0270 (3)	
H18A	0.4866	0.6189	0.6025	0.032*	
H18B	0.3167	0.5519	0.6283	0.032*	
C19	0.43686 (17)	0.76375 (15)	0.69446 (8)	0.0334 (3)	
H19A	0.5066	0.8523	0.6845	0.050*	
H19B	0.5018	0.7313	0.7385	0.050*	
H19C	0.3360	0.7861	0.7100	0.050*	
C20	0.41197 (14)	0.68357 (12)	0.17361 (7)	0.0182 (3)	
H20A	0.3521	0.7167	0.2153	0.022*	
H20B	0.5329	0.7351	0.1940	0.022*	
C21	0.39391 (14)	0.51959 (13)	0.16401 (7)	0.0199 (3)	
H21A	0.4560	0.5013	0.2147	0.024*	
H21B	0.2733	0.4694	0.1557	0.024*	
C22	0.46880 (13)	0.81199 (12)	0.06247 (7)	0.0172 (3)	
H22A	0.4215	0.7940	0.0033	0.021*	
H22B	0.5732	0.7769	0.0703	0.021*	
C23	0.51254 (14)	0.97586 (12)	0.09878 (7)	0.0202 (3)	
H23A	0.5500	0.9930	0.1586	0.024*	
H23B	0.6078	1.0255	0.0784	0.024*	
H5	−0.3060 (10)	0.5149 (14)	0.0952 (7)	0.033 (4)*	
H6	−0.2096 (11)	0.8076 (14)	−0.0843 (7)	0.031 (4)*	

### Atomic displacement parameters (Å^2^)

**Table:**

	*U*^11^	*U*^22^	*U*^33^	*U*^12^	*U*^13^	*U*^23^
O1	0.0150 (4)	0.0274 (5)	0.0234 (5)	0.0047 (3)	0.0012 (3)	0.0000 (4)
O2	0.0178 (4)	0.0190 (5)	0.0363 (6)	0.0049 (3)	0.0051 (4)	0.0037 (4)
N1	0.0133 (5)	0.0196 (5)	0.0164 (5)	0.0037 (4)	0.0030 (4)	0.0053 (4)
N2	0.0142 (5)	0.0187 (5)	0.0153 (5)	0.0040 (4)	0.0029 (4)	0.0039 (4)
N3	0.0132 (5)	0.0178 (5)	0.0157 (5)	0.0044 (4)	0.0016 (4)	0.0045 (4)
N4	0.0126 (5)	0.0205 (5)	0.0176 (5)	0.0026 (4)	0.0022 (4)	0.0084 (4)
N5	0.0134 (5)	0.0236 (6)	0.0198 (6)	0.0042 (4)	0.0037 (4)	0.0077 (5)
N6	0.0133 (5)	0.0212 (6)	0.0174 (5)	0.0053 (4)	0.0021 (4)	0.0069 (4)
C1	0.0157 (6)	0.0143 (6)	0.0143 (6)	0.0037 (4)	0.0025 (5)	0.0001 (5)
C2	0.0146 (6)	0.0154 (6)	0.0144 (6)	0.0030 (4)	0.0029 (5)	0.0003 (5)
C3	0.0166 (6)	0.0141 (6)	0.0140 (6)	0.0049 (4)	0.0025 (5)	−0.0001 (5)
C4	0.0181 (6)	0.0159 (6)	0.0164 (6)	0.0032 (4)	0.0022 (5)	0.0043 (5)
C5	0.0197 (6)	0.0249 (7)	0.0179 (7)	0.0055 (5)	0.0036 (5)	0.0064 (5)
C6	0.0253 (7)	0.0235 (7)	0.0188 (7)	0.0025 (5)	0.0032 (5)	0.0058 (5)
C7	0.0311 (7)	0.0227 (7)	0.0214 (7)	0.0064 (5)	0.0055 (5)	0.0083 (6)
C8	0.0316 (7)	0.0255 (7)	0.0201 (7)	0.0104 (5)	0.0055 (5)	0.0073 (6)
C9	0.0331 (7)	0.0294 (7)	0.0212 (7)	0.0123 (6)	0.0056 (6)	0.0075 (6)
C10	0.0393 (8)	0.0371 (8)	0.0213 (7)	0.0171 (6)	0.0021 (6)	0.0028 (6)
C11	0.0565 (10)	0.0616 (11)	0.0225 (8)	0.0256 (8)	−0.0021 (7)	0.0042 (8)
C12	0.0189 (6)	0.0204 (6)	0.0207 (7)	0.0034 (5)	0.0064 (5)	0.0079 (5)
C13	0.0245 (6)	0.0225 (7)	0.0202 (7)	0.0079 (5)	0.0088 (5)	0.0070 (5)
C14	0.0251 (6)	0.0230 (7)	0.0224 (7)	0.0074 (5)	0.0073 (5)	0.0072 (6)
C15	0.0300 (7)	0.0253 (7)	0.0210 (7)	0.0104 (5)	0.0056 (5)	0.0061 (6)
C16	0.0266 (7)	0.0258 (7)	0.0218 (7)	0.0101 (5)	0.0058 (5)	0.0067 (6)
C17	0.0287 (7)	0.0261 (7)	0.0210 (7)	0.0096 (5)	0.0048 (5)	0.0058 (6)
C18	0.0273 (7)	0.0297 (7)	0.0240 (7)	0.0086 (5)	0.0041 (5)	0.0064 (6)
C19	0.0340 (8)	0.0378 (9)	0.0247 (8)	0.0081 (6)	0.0003 (6)	0.0054 (7)
C20	0.0146 (6)	0.0221 (7)	0.0165 (6)	0.0036 (5)	−0.0003 (5)	0.0059 (5)
C21	0.0174 (6)	0.0241 (7)	0.0193 (7)	0.0057 (5)	0.0030 (5)	0.0082 (5)
C22	0.0130 (5)	0.0209 (6)	0.0194 (6)	0.0045 (4)	0.0044 (5)	0.0074 (5)
C23	0.0168 (6)	0.0209 (7)	0.0222 (7)	0.0030 (5)	0.0030 (5)	0.0063 (5)

### Geometric parameters (Å, º)

**Table:**

O1—C21	1.4224 (14)	C10—H10B	0.9900
O1—H1	0.8400	C11—H11A	0.9800
O2—C23	1.4275 (13)	C11—H11B	0.9800
O2—H2	0.8400	C11—H11C	0.9800
N1—C1	1.3386 (14)	C12—C13	1.5217 (16)
N1—C2	1.3433 (14)	C12—H12A	0.9900
N2—C3	1.3507 (14)	C12—H12B	0.9900
N2—C2	1.3521 (14)	C13—C14	1.5240 (15)
N3—C3	1.3431 (14)	C13—H13A	0.9900
N3—C1	1.3583 (14)	C13—H13B	0.9900
N4—C1	1.3522 (14)	C14—C15	1.5203 (17)
N4—C20	1.4619 (14)	C14—H14A	0.9900
N4—C22	1.4666 (14)	C14—H14B	0.9900
N5—C2	1.3407 (14)	C15—C16	1.5196 (16)
N5—C12	1.4557 (14)	C15—H15A	0.9900
N5—H5	0.893 (8)	C15—H15B	0.9900
N6—C3	1.3497 (14)	C16—C17	1.5242 (17)
N6—C4	1.4557 (14)	C16—H16A	0.9900
N6—H6	0.895 (8)	C16—H16B	0.9900
C4—C5	1.5135 (15)	C17—C18	1.5211 (16)
C4—H4A	0.9900	C17—H17A	0.9900
C4—H4B	0.9900	C17—H17B	0.9900
C5—C6	1.5245 (16)	C18—C19	1.5223 (18)
C5—H5A	0.9900	C18—H18A	0.9900
C5—H5B	0.9900	C18—H18B	0.9900
C6—C7	1.5259 (16)	C19—H19A	0.9800
C6—H6A	0.9900	C19—H19B	0.9800
C6—H6B	0.9900	C19—H19C	0.9800
C7—C8	1.5179 (17)	C20—C21	1.5138 (17)
C7—H7A	0.9900	C20—H20A	0.9900
C7—H7B	0.9900	C20—H20B	0.9900
C8—C9	1.5257 (16)	C21—H21A	0.9900
C8—H8A	0.9900	C21—H21B	0.9900
C8—H8B	0.9900	C22—C23	1.5141 (16)
C9—C10	1.5104 (18)	C22—H22A	0.9900
C9—H9A	0.9900	C22—H22B	0.9900
C9—H9B	0.9900	C23—H23A	0.9900
C10—C11	1.5213 (17)	C23—H23B	0.9900
C10—H10A	0.9900		
			
C21—O1—H1	109.5	N5—C12—H12A	109.1
C23—O2—H2	109.5	C13—C12—H12A	109.1
C1—N1—C2	114.52 (10)	N5—C12—H12B	109.1
C3—N2—C2	113.97 (9)	C13—C12—H12B	109.1
C3—N3—C1	114.02 (9)	H12A—C12—H12B	107.8
C1—N4—C20	120.28 (10)	C12—C13—C14	114.34 (10)
C1—N4—C22	121.39 (9)	C12—C13—H13A	108.7
C20—N4—C22	117.74 (9)	C14—C13—H13A	108.7
C2—N5—C12	121.87 (9)	C12—C13—H13B	108.7
C2—N5—H5	120.2 (8)	C14—C13—H13B	108.7
C12—N5—H5	117.9 (8)	H13A—C13—H13B	107.6
C3—N6—C4	123.16 (9)	C15—C14—C13	111.74 (10)
C3—N6—H6	116.9 (8)	C15—C14—H14A	109.3
C4—N6—H6	118.5 (8)	C13—C14—H14A	109.3
N1—C1—N4	116.90 (10)	C15—C14—H14B	109.3
N1—C1—N3	125.73 (10)	C13—C14—H14B	109.3
N4—C1—N3	117.35 (10)	H14A—C14—H14B	107.9
N5—C2—N1	116.81 (10)	C16—C15—C14	116.01 (10)
N5—C2—N2	117.38 (9)	C16—C15—H15A	108.3
N1—C2—N2	125.81 (10)	C14—C15—H15A	108.3
N3—C3—N6	117.08 (10)	C16—C15—H15B	108.3
N3—C3—N2	125.91 (10)	C14—C15—H15B	108.3
N6—C3—N2	117.00 (9)	H15A—C15—H15B	107.4
N6—C4—C5	111.51 (9)	C15—C16—C17	112.37 (10)
N6—C4—H4A	109.3	C15—C16—H16A	109.1
C5—C4—H4A	109.3	C17—C16—H16A	109.1
N6—C4—H4B	109.3	C15—C16—H16B	109.1
C5—C4—H4B	109.3	C17—C16—H16B	109.1
H4A—C4—H4B	108.0	H16A—C16—H16B	107.9
C4—C5—C6	112.08 (9)	C18—C17—C16	115.39 (11)
C4—C5—H5A	109.2	C18—C17—H17A	108.4
C6—C5—H5A	109.2	C16—C17—H17A	108.4
C4—C5—H5B	109.2	C18—C17—H17B	108.4
C6—C5—H5B	109.2	C16—C17—H17B	108.4
H5A—C5—H5B	107.9	H17A—C17—H17B	107.5
C5—C6—C7	113.74 (10)	C17—C18—C19	112.27 (11)
C5—C6—H6A	108.8	C17—C18—H18A	109.1
C7—C6—H6A	108.8	C19—C18—H18A	109.1
C5—C6—H6B	108.8	C17—C18—H18B	109.1
C7—C6—H6B	108.8	C19—C18—H18B	109.2
H6A—C6—H6B	107.7	H18A—C18—H18B	107.9
C8—C7—C6	115.39 (10)	C18—C19—H19A	109.5
C8—C7—H7A	108.4	C18—C19—H19B	109.5
C6—C7—H7A	108.4	H19A—C19—H19B	109.5
C8—C7—H7B	108.4	C18—C19—H19C	109.5
C6—C7—H7B	108.4	H19A—C19—H19C	109.5
H7A—C7—H7B	107.5	H19B—C19—H19C	109.5
C7—C8—C9	113.02 (10)	N4—C20—C21	114.49 (9)
C7—C8—H8A	109.0	N4—C20—H20A	108.6
C9—C8—H8A	109.0	C21—C20—H20A	108.6
C7—C8—H8B	109.0	N4—C20—H20B	108.6
C9—C8—H8B	109.0	C21—C20—H20B	108.6
H8A—C8—H8B	107.8	H20A—C20—H20B	107.6
C10—C9—C8	113.91 (11)	O1—C21—C20	112.63 (10)
C10—C9—H9A	108.8	O1—C21—H21A	109.1
C8—C9—H9A	108.8	C20—C21—H21A	109.1
C10—C9—H9B	108.8	O1—C21—H21B	109.1
C8—C9—H9B	108.8	C20—C21—H21B	109.1
H9A—C9—H9B	107.7	H21A—C21—H21B	107.8
C9—C10—C11	113.88 (11)	N4—C22—C23	112.52 (10)
C9—C10—H10A	108.8	N4—C22—H22A	109.1
C11—C10—H10A	108.8	C23—C22—H22A	109.1
C9—C10—H10B	108.8	N4—C22—H22B	109.1
C11—C10—H10B	108.8	C23—C22—H22B	109.1
H10A—C10—H10B	107.7	H22A—C22—H22B	107.8
C10—C11—H11A	109.5	O2—C23—C22	112.85 (9)
C10—C11—H11B	109.5	O2—C23—H23A	109.0
H11A—C11—H11B	109.5	C22—C23—H23A	109.0
C10—C11—H11C	109.5	O2—C23—H23B	109.0
H11A—C11—H11C	109.5	C22—C23—H23B	109.0
H11B—C11—H11C	109.5	H23A—C23—H23B	107.8
N5—C12—C13	112.69 (10)		
			
C2—N1—C1—N4	179.73 (10)	C3—N6—C4—C5	162.91 (10)
C2—N1—C1—N3	1.35 (16)	N6—C4—C5—C6	−179.78 (10)
C20—N4—C1—N1	−10.55 (15)	C4—C5—C6—C7	−176.09 (10)
C22—N4—C1—N1	178.45 (10)	C5—C6—C7—C8	−67.98 (15)
C20—N4—C1—N3	167.96 (10)	C6—C7—C8—C9	179.15 (10)
C22—N4—C1—N3	−3.03 (15)	C7—C8—C9—C10	178.89 (11)
C3—N3—C1—N1	−2.26 (16)	C8—C9—C10—C11	179.31 (12)
C3—N3—C1—N4	179.37 (10)	C2—N5—C12—C13	77.29 (14)
C12—N5—C2—N1	−1.91 (16)	N5—C12—C13—C14	−165.17 (10)
C12—N5—C2—N2	177.83 (9)	C12—C13—C14—C15	177.18 (10)
C1—N1—C2—N5	179.59 (10)	C13—C14—C15—C16	179.77 (10)
C1—N1—C2—N2	−0.12 (16)	C14—C15—C16—C17	−178.44 (11)
C3—N2—C2—N5	−179.72 (10)	C15—C16—C17—C18	178.60 (11)
C3—N2—C2—N1	−0.01 (16)	C16—C17—C18—C19	−177.13 (11)
C1—N3—C3—N6	−177.87 (9)	C1—N4—C20—C21	77.14 (13)
C1—N3—C3—N2	2.11 (16)	C22—N4—C20—C21	−111.54 (11)
C4—N6—C3—N3	−1.14 (16)	N4—C20—C21—O1	50.37 (13)
C4—N6—C3—N2	178.88 (10)	C1—N4—C22—C23	85.59 (12)
C2—N2—C3—N3	−1.08 (16)	C20—N4—C22—C23	−85.63 (12)
C2—N2—C3—N6	178.90 (9)	N4—C22—C23—O2	−67.59 (13)

### Hydrogen-bond geometry (Å, º)

**Table:**

*D*—H···*A*	*D*—H	H···*A*	*D*···*A*	*D*—H···*A*
O1—H1···N2^i^	0.84	1.95	2.7732 (13)	167
O2—H2···N3	0.84	2.00	2.8057 (14)	160
N5—H5···O1^ii^	0.89 (1)	1.95 (1)	2.7963 (15)	157 (1)
N6—H6···O2^iii^	0.90 (1)	2.20 (1)	2.9455 (14)	141 (1)

Symmetry codes: (i) −*x*, −*y*+1, −*z*; (ii) *x*−1, *y*, *z*; (iii) −*x*, −*y*+2, −*z*.
